# No Excess Cancer Risk among Veterinarians in Denmark, Finland, Iceland, Norway, and Sweden after the 1980s

**DOI:** 10.3390/cancers15164079

**Published:** 2023-08-13

**Authors:** Laura Laakso, Pikka Jokelainen, Hans Houe, Eystein Skjerve, Johnni Hansen, Elsebeth Lynge, Jan-Ivar Martinsen, Ingrid Sivesind Mehlum, Jenny Selander, Jóhanna Eyrún Torfadóttir, Elisabete Weiderpass, Sanna Heikkinen, Eero Pukkala

**Affiliations:** 1 Animal Clinic of Paippinen, 04170 Paippinen, Finland; laura.laakso@equidea.fi; 2 Infectious Disease Preparedness, Statens Serum Institut, 2300 Copenhagen, Denmark; 3 Faculty of Veterinary Medicine, University of Helsinki, 00014 Helsinki, Finland; 4 Department of Veterinary and Animal Sciences, University of Copenhagen, 1870 Frederiksberg, Denmark; houe@sund.ku.dk; 5 The Faculty of Veterinary Medicine, Norwegian University of Life Sciences, 1433 Ås, Norway; eystein.skjerve@nmbu.no; 6 Danish Cancer Society Research Center, Danish Cancer Society, 2100 Copenhagen, Denmark; johnni@cancer.dk; 7 Nykøbing Falster Hospital and Department of Public Health, University of Copenhagen, 1014 Copenhagen, Denmark; elsebeth@sund.ku.dk; 8 Department of Research, Cancer Registry of Norway, 0379 Oslo, Norway; jan.ivar.martinsen@kreftregisteret.no; 9 Department of Occupational Medicine and Epidemiology, National Institute of Occupational Health (STAMI), 0304 Oslo, Norway; ingrid.s.mehlum@stami.no; 10 Unit of Occupational Medicine, Institute of Environmental Medicine, 171 77 Stockholm, Sweden; jenny.selander@ki.se; 11 Department of Education & Prevention, The Icelandic Cancer Society, 105 Reykjavik, Iceland; jet@hi.is; 12 Centre for Public Health Sciences, University of Iceland, 102 Reykjavik, Iceland; 13 International Agency for Research on Cancer (IARC), World Health Organization, 69372 Lyon, France; weiderpasse@iarc.fr; 14 Finnish Cancer Registry, Institute for Statistical and Epidemiological Cancer Research, 00130 Helsinki, Finland; sanna.heikkinen@cancer.fi; 15 Health Sciences Unit, Faculty of Social Sciences, Tampere University, 33520 Tampere, Finland

**Keywords:** cancer, cohort, Nordic countries, occupational health, veterinary profession

## Abstract

**Disclaimer:**

Where authors are identified as personnel of the International Agency for Research on Cancer/World Health Organization, the authors alone are responsible for the views expressed in this article and they do not necessarily represent the decisions, policy, or views of the International Agency for Research on Cancer/World Health Organization.

**Simple Summary:**

Veterinarians can be exposed to a wide range of known and suspected carcinogens through their work, however relatively few studies have investigated the cancer risk in the profession. We investigated cancer incidence in veterinarians in Denmark, Finland, Iceland, Norway, and Sweden, across more than 40 years. In all the countries, the overall cancer incidence in veterinarians was close to the incidence in the total population. There was an elevated incidence of several cancer types among male veterinarians before the 1990s but no excess cancer risk after that. Veterinary work environments have been changing, and are changing, in terms of exposure to chemical compounds, ionizing radiation from diagnostic imaging, and different pathogens, and these changes may affect the cancer profile of veterinarians.

**Abstract:**

The cancer profile of veterinarians has received little research attention, despite the profession potentially being exposed to a wide range of known and suspected carcinogens. In this large-scale cohort study, we assessed cancer incidence in veterinarians in Denmark, Finland, Iceland, Norway, and Sweden, across more than 40 years (1961–2005). The cohort comprised 4708 veterinarians and 119,503 person-years at follow-up. The overall cancer incidence in veterinarians was close to the incidence in the total population in all countries and in all age groups. In male veterinarians, the standardized incidence ratios (SIR) in 1961–1990 were elevated for colon cancer (1.86, 95% confidence interval (CI) 1.39–2.44), prostate cancer (1.35, 95% CI 1.07–1.67), and especially skin melanoma (3.62, 95% CI 2.78–2.84), while there was no longer any statistically significant excess in the more recent follow-up period. Decreased SIRs were observed for lip cancer (0.11, 95% CI 0.00–0.62), laryngeal cancer (0.38, 95% CI 0.12–0.89), lung cancer (0.59, 95% CI 0.47–0.74), and stomach cancer (0.58, 95% CI 0.38–0.86), without a marked change in SIR over time. Non-significant excesses among male veterinarians were also observed in Hodgkin lymphoma (1961–1990 only), and leukaemia. This multi-country study indicates that there was an elevated incidence of several cancer types among male veterinarians before the 1990s but not after that. Some of the findings might rather be attributed to lifestyle factors and not directly to work conditions, but the excess risk of cancers of kidney and bladder, for example, might be related to work exposures.

## 1. Introduction

Veterinarians are potentially exposed to a wide range of known and suspected carcinogens through their work. However, relatively few studies have investigated the cancer risk in veterinarians [1,2,3,4,5,6,7].

The work of veterinarians includes a wide variety of work types, from clinical practice to laboratory and office work. Potential risk factors for cancer due to veterinary work include ionizing radiation, chemical compounds, organic dusts, infectious agents including zoonotic pathogens, stressful work, and working in night shifts. The exposure to different chemical compounds is in constant change, as new drugs are introduced to the veterinary market [8]. Veterinarians are also exposed to a wide range of micro-organisms, including both endemic and emerging zoonotic pathogens [9,10,11,12,13], and if such pathogens would cause cancer in humans, veterinarians could be one of the first professions to show the signs of such risks. In addition to the directly work-related risks, the background risks of a high socioeconomic position need to be considered when investigating the cancer profile of the veterinary profession. 

In this study, we assessed the incidence of cancer in a cohort of veterinarians in the five Nordic countries in comparison to their entire national populations in order to identify any excess or deficit cancer risks.

## 2. Materials and Methods

This cohort study was based on data of the Nordic Occupational Cancer (NOCCA) project where a linkage was built between the census data for 15 million individuals living in the Nordic countries (Denmark, Finland, Iceland, Norway, and Sweden) and the national cancer registry data from 1961 to 2005 [14]. These countries have a long tradition of high-quality cancer registrations with relatively small differences between the countries in, for example, data sources and cancer coding [15]. Data linkages were made utilizing national unique personal identity codes.

The cohort of veterinarians was defined by identifying veterinarians from the coding systems using information on education, occupation, industry, and employer at the time of the census. To avoid misclassification related to the beginning of the work career, we considered the occupation from the age of 30 years or older. A person entered the cohort on January 1st of the year after the first available census, provided they were a veterinarian, 30–64 years old, and living in the country. Census records were available from Denmark from 1970; from Finland from 1970, 1980, and 1990; from Iceland from 1981; from Norway from 1960, 1970, and 1980; and from Sweden from 1960, 1970, 1980, and 1990.

Person-years were counted from the date of entry to the date of emigration, death, or to 31 December of the following years: in Denmark 2003, in Finland 2005, in Iceland 2004, in Norway 2003, and in Sweden 2005. The data on dates of death and emigration were extracted from the national population registries.

All incident cancer cases were included. The cancer cases were grouped into 49 main categories and 27 diagnostic subgroups based on topography and morphology coding systems. We examined the patterns of cancer incidence among the veterinarians by gender, country, time period, and age at follow-up.

We report the relative levels of the incidence of cancers among the veterinarians as standardized incidence ratios (SIR), using the cancer incidence for the respective national populations as reference. For each country and gender, the observed and expected numbers of cancer cases were calculated by five-year categories of age at follow-up and by five-year calendar time periods. For presentation purposes we divided the observed and expected numbers of cancer cases into two time periods (1961–1990, and 1991–2005). The SIR was then calculated as a ratio of the observed number and expected number of cancer cases. For each SIR, the exact 95% confidence interval (CI) was calculated, assuming a Poisson distribution of the observed number of cases. 

## 3. Results

The cohort comprised 4708 veterinarians, 83% men and 17% women (Table 1). The total number of person-years at follow-up was 119,503. 

The overall cancer incidence in veterinarians of either gender was not statistically significantly different from the reference incidence (Table 2). In male veterinarians, cancer incidence in 1961–1990 was however 14% higher than in the reference population. None of the country-specific SIRs were significantly different from 1.0. 

The SIRs for cancer of the colon, urinary organs and prostate, and for skin melanoma were significantly elevated before the 1990s but not in 1991–2005 (Table 3). The SIR for urinary cancers in the latter observation period was 0.64 (95% CI 0.46–0.84). SIRs significantly below 1.0 were observed for cancers of the stomach, lung, larynx, and lip; these SIRs did not markedly change between the observation periods (Table 3).

The six cases of Hodgkin lymphoma diagnosed before 1990 raised the SIR to 2.51 (95% CI 0.93–5.49). Three of the cases were from Finland (SIR 6.90, 95% CI 1.42–20.2).

## 4. Discussion

This is the first large-scale multi-country cohort study on cancer incidence among veterinarians. Veterinarians are a relatively small profession and have often been categorized as, for example, ‘Other health and medical workers’ [14,16]. In this study, the multi-country study design and long follow-up time enabled a focus on the veterinary profession. The study showed that veterinarians had an overall cancer risk similar to that of the background population but there were several cancer sites with elevated incidence levels among male veterinarians before the 1990s.

Many of the potential occupational risks for the veterinary profession are similar to those of medical doctors, but exposure levels and the degree of control of the related occupational hazards may differ. The cancer profile of the veterinary profession could highlight occupational hazards of relevance to other professions including medical doctors, dentists, veterinary nurses, laboratory personnel, abattoir workers, and farmers [6]. 

Although an occupation at one point in time may not correctly reflect the lifelong occupational history of an individual, occupational stability among veterinarians is high and the bias due to misclassification of occupation is thus considered negligible. As the present study was based on incident cancer cases and exact person-years, there was no bias caused by occupational variation in cancer survival or in mortality from competing causes of death, which may be a challenge in analyses based on cancer deaths and cross-sectional proportionate analyses.

Until 1990, male veterinarians had a strongly elevated SIR for skin melanoma which is known to be associated with sunburns and to be typical for indoor workers whose skin is not used to regular sun exposure [17]. This could be due to lifestyle factors and not directly linked to work conditions; traveling to sunny places was more possible for high-income groups during the earlier years of the follow-up but became available to larger population groups in later years. The finding is in line with the recently reported higher than expected proportionate mortality from malignant melanoma among veterinarian decedents in 1979–2015 in the USA [18]. The authors of that study encouraged enforcing occupational health and safety guidelines for sun exposure as well as radiology safety protocols to reduce the number of deaths from malignant melanoma among veterinarians. The pattern of decreased SIR for stomach cancer in combination with an increased SIR for colon cancer is typical for higher socioeconomic classes [19,20], and could indicate differences in dietary patterns [21]. Moreover, higher socioeconomic status has been linked with a higher risk of prostate cancer probably due to greater access to private prostate-specific antigen (PSA) testing [22,23]. The effect of private screening to the occupational incidence variation of other cancers is expected to be limited. There have been organized screening programs for cervical cancer which started in the 1960s in Finland, Iceland, and Sweden and later in Norway and Denmark. Organised mammography screenings started in the late 1980s in Sweden, Finland, and Iceland. Screenings are offered to all, and their effect on incidence is therefore expected to be similar across professions. The possible effect of selective screening participation among veterinarians on the risk of cervical and breast cancer cannot be estimated because the numbers of these cancers among female veterinarians are very small. Screening for colorectal cancer in the Nordic countries began after the follow-up period of the present study.

The low incidence of lung cancer and laryngeal cancer in both follow-up periods suggests that male veterinarians smoked less than men in general. With that perspective, the elevated risk of cancers of the kidney and urinary bladder in the first follow-up period is unexpected because smoking is also one of the risk factors of these cancers. There is sufficient evidence of a relationship between exposure to X-ray radiation, gamma radiation, and trichloroethylene, and the increased risk of renal tumours [24], and increased risk of bladder cancer has been reported from workplace exposure to solvents such as tetrachloroethylene and o-toluidine [25]. It is not quite impossible that veterinarians in the past had some of these exposures.

Exposure to ionizing radiation from diagnostic imaging procedures has been estimated to be low in veterinarians [26]. Nevertheless, the observed slight, non-significant increase in leukaemia, although based on relatively few cases, might warrant further studies. Because exposure to ionizing radiation varies by type of work, sub-grouping within the profession could be useful for investigating this further. This was unfortunately not possible in this study, as more specific job titles were not available.

The excess Hodgkin lymphoma cases in male veterinarians diagnosed before 1990 might suggest a potentially infection-related aetiology [1,27,28]. The time-dependency suggests the exposure being prevented or becoming rare. The strengths of our study include the multi-country cohort design with a long follow-up and high-quality register data. Nordic countries are well known for their long tradition of high-quality population-based cancer registration. Any inaccuracies in cancer registration are not expected to affect the SIR estimates of the present study as they are unrelated to occupation. The study was a large-scale study, however, the low number of cases in women was a limitation. Nowadays women comprise a much larger proportion of the veterinary profession in the Nordic countries.

No major cancer risk specific to the veterinary profession was identified in the most recent time period of this study. There was an elevated incidence of several cancer types among male veterinarians before the 1990s but not after that. While many of the detected deviations from the national cancer patterns probably reflected lifestyle more than work-related exposures, the results provided a unique baseline and enable the formulating of relevant research questions for future studies. It would help in separating the effects of socio-economic status (SES) and other factors if we could have access to SES-specific reference rates but unfortunately such rates are not available for the Nordic countries during the study period. If we could have used SES-specific reference incidence rates, then the SIR estimates would be higher for cancer types that are more common in low-SES populations (e.g., stomach and lung), and lower for cancer types that are more common in high-SES populations (e.g., skin melanoma and colon).

Our results support the conclusion of the recent study from the USA [18]—certain cancers among veterinarians merit further studies, and mitigation strategies should be considered. A comprehensive review of potential carcinogenic exposures specific to selected professions, including veterinarians, would be useful. 

Some local information material about the occupational health of veterinarians is available (e.g., [29]), and such material should be regularly updated. Veterinary work environments have been changing and are changing in terms of exposure to chemical compounds, ionizing radiation from diagnostic imaging, zoonotic pathogens, and awareness of occupational hazards, and these changes may affect the future cancer profile of veterinarians.

## 5. Conclusions

The overall cancer incidence in veterinarians was close to the incidence in the total population. There was an elevated incidence of several cancer types among male veterinarians before the 1990s but no excess cancer risk after that. 

## Figures and Tables

**Table 1:** Veterinarians included in the Nordic Occupational Cancer (NOCCA) study cohort: number of persons and person-years, by gender and country.

Country	Men		Women	
	Persons	Person-Years	Persons	Person-Years
Denmark	1277	32,510	28	884
Finland	550	13,798	295	5746
Iceland	28	581	1	23
Norway	787	21,864	43	1155
Sweden	1256	34,626	443	8316
Total	3898	103,379	810	16,124

**Table 2:** Observed numbers (*n*) of cancer cases and standardized incidence ratios (SIR) with 95% confidence intervals (CI) among veterinarians in the Nordic Occupational Cancer Study (NOCCA) cohort, by gender, time period, and country.

Category	Men			Women		
	*n*	SIR	95% CI	*n*	SIR	95% CI
Time period						
1961–1990	426	1.14	1.04–1.26	10	0.74	0.35–1.36
1991–2005	435	0.86	0.78–0.95	44	0.86	0.62–1.15
Country						
Denmark	335	0.92	0.82–1.03	5	0.67	0.21–1.56
Finland	98	1.17	0.95–1.43	16	0.79	0.45–1.28
Iceland	4	0.90	0.25–2.30	0	0.00	0.00–41.10
Norway	161	0.94	0.80–1.10	7	1.16	0.47–2.39
Sweden	263	1.03	0.90–1.16	26	0.84	0.54–1.23
Total	861	0.98	0.92–1.05	54	0.83	0.63–1.09

**Table 3:** Observed numbers (*n*) of cancer cases and standardized incidence ratios (SIR) of cancer, with 95% confidence intervals (CI), among male veterinarians in the Nordic Occupational Cancer Study (NOCCA) cohort, by time period and cancer type.

Cancer Type	1961–1990			1991–2005			Total		
	*n*	SIR	95% CI	*n*	SIR	95% CI	*n*	SIR	95% CI
Stomach	17	0.64	0.37–1.03	8	0.48	0.21–0.95	25	0.58	0.38–0.86
Colon	52	1.86	1.39–2.44	48	1.07	0.79–1.42	100	1.38	1.12–1.67
Lung	45	0.62	0.45–0.83	39	0.56	0.40–0.77	84	0.59	0.47–0.74
Lip	0	0.00	0.00–0.95	1	0.20	0.01–1.11	1	0.11	0.00–0.62
Larynx	3	0.53	0.11–1.56	2	0.27	0.03–0.97	5	0.38	0.12–0.89
Urinary organs	58	1.39	1.06–1.80	42	0.64	0.41–0.85	100	1.02	0.83–1.24
kidney	21	1.49	0.92–2.28	11	0.76	0.38–1.37	32	1.12	0.77–1.58
bladder	37	1.34	0.94–1.84	31	0.60	0.41–0.85	68	0.85	0.66–1.08
Prostate	82	1.35	1.07–1.67	145	1.12	0.94–1.31	227	1.19	1.04–1.35
Skin melanoma	32	3.62	2.78–5.11	23	1.41	0.89–2.11	55	2.18	1.65–2.84
Hodgkin lymphoma	6	2.51	0.93–5.49	0	0.00	0.00–2.52	6	1.56	0.57–3.93
Leukaemia	13	1.23	0.65–2.10	17	1.30	0.76–2.08	30	1.26	0.85–1.80
chronic lymphatic	6	2.54	0.93–5.53	2	0.68	0.08–2.44	8	1.50	0.65–2.96
acute myeloid	3	0.93	0.19–2.72	7	2.05	0.83–4.23	10	1.51	0.72–2.78

## Data Availability

See the Nordic Occupational Cancer Study (NOCCA) website https://astra.cancer.fi/NOCCA/ (accessed on 10 August 2023) for instructions. The tabulated data used as the basis of this study are available on request from the corresponding authors. Individual-level data are only available if they are reconstructed from the original source registries.
